# Detection of Ehrlichia spp. and Theileria spp. in Hyalomma anatolicum ticks collected in Tajikistan

**DOI:** 10.18699/VJ20.595

**Published:** 2020-02

**Authors:** M.Yu. Kartashov, Yu.V. Kononova, I.D. Petrova, N.L. Tupota, T.P. Mikryukova, V.A. Ternovoi, F.H. Tishkova, V.B. Loktev

**Affiliations:** State Research Center for Virology and Biotechnology “Vector”, Koltsovo, Novosibirsk region, Russia Novosibirsk State University, Novosibirsk, Russia; State Research Center for Virology and Biotechnology “Vector”, Koltsovo, Novosibirsk region, Russia Tomsk State University, Tomsk, Russia; State Research Center for Virology and Biotechnology “Vector”, Koltsovo, Novosibirsk region, Russia; State Research Center for Virology and Biotechnology “Vector”, Koltsovo, Novosibirsk region, Russia; State Research Center for Virology and Biotechnology “Vector”, Koltsovo, Novosibirsk region, Russia Tomsk State University, Tomsk, Russia; State Research Center for Virology and Biotechnology “Vector”, Koltsovo, Novosibirsk region, Russia Tomsk State University, Tomsk, Russia; Tajik Research Institute of Preventive Medicine, Dushanbe, Tajikistan; State Research Center for Virology and Biotechnology “Vector”, Koltsovo, Novosibirsk region, Russia Novosibirsk State University, Novosibirsk, Russia Tomsk State University, Tomsk, Russia Institute of Cytology and Genetics of Siberian Branch of the Russian Academy of Sciences, Novosibirsk, Russia

**Keywords:** Hyalomma anatolicum, tick-borne infections, Ehrlichia spp., Theileria spp., Tajikistan, Hyalomma anatolicum, клещевые инфекции, Ehrlichia spp., Theileria spp., Таджикистан

## Abstract

The objectives of our study were to survey the prevalence of genetic markers for Rickettsia spp., Ehrlichia spp., Anaplasma spp., Babesia spp., and Theileria spp. in Hyalomma anatolicum ticks collected in southwestern Tajikistan and to perform sequencing and phylogenetic analysis of fragments of the 16S rRNA gene and groESL operon from Ehrlichia spp. and fragments of the 18S rRNA gene of Theileria spp. detected in H. anatolicum ticks. Hyalomma anatolicum ticks collected in the Tursunzade and Rudaki districts of Tajikistan were tested for DNA of Rickettsia spp., Ehrlichia spp., Anaplasma spp., Babesia spp., and Theileria spp. by PCR with specific primers. The amplified fragments were sequenced and analyzed. DNA of Ehrlichia spp. (3.3 %) and Theileria spp. (3.3 %) was detected only in H. anatolicum ticks collected from the Rudaki district, and DNA of Ehrlichia spp. (0.7 %) was found in H. anatolicum ticks from the Tursunzade district. Sequence analysis of fragments of the 16S rRNA gene and groESL operon from Ehrlichia spp. revealed high similarity to Ehrlichia spp. The Tajik isolates of Theileria spp. were genotyped as Theileria annulata based on the analysis of 18S rRNA gene sequences. The phylogenetic analysis demonstrates that Ehrlichia spp. isolates are highly similar to Ehrlichia spp. circulating in China and Brazil. The isolate Tajikistan-5 is closely related to the putative novel species Ehrlichia mineirensis. The Tajik isolates of Theileria spp. were clustered with T. annulata isolates from Turkey, Iran, Pakistan, and China by phylogenetic analyses.

## Abstract (ru)

Исследовано наличие генетического материала Rickettsia spp., Ehrlichia spp., Anaplasma spp., Babesia spp. и Theileria spp. в клещах Hyalomma anatolicum, собранных в юго-западной части Таджикистана, с последующим секвенированием и филогенетическим анализом фрагментов 16S рРНК гена и groESL оперона для Ehrlichia spp. и фрагмента гена 18S рРНК для Theileria spp., обнаруженного в изученных клещах. Клещи H. anatolicum были собраны в районах Турсунзаде и Рудаки и исследованы с использованием специфичных праймеров c помощью ПЦР на наличие генетического материала Rickettsia spp., Ehrlichia spp., Anaplasma spp., Babesia spp. и Theileria spp. Выделенные ПЦР-фрагменты генов 16S рРНК, groESL оперона и 18S рРНК были секвенированы и проведен их филогенетический анализ с целью генотипирования обнаруженных изолятов клещевых патогенов. В клещах, собранных в районе Рудаки, обнаружена ДНК Ehrlichia spp. (3.3 %) и Theileria spp. (3.3 %), а в Турсунзаде – ДНК Ehrlichia spp. (0.7 %). Секвенирование фрагментов гена 16S рРНК и groESL оперона Ehrlichia spp. показало высокий уровень гомологии нуклеотидной последовательности с известными последовательностями Ehrlichia spp. Таджикские изоляты Theileria spp. были генотипированы как Theileria annulata на основе анализа последовательности гена 18S рРНК. Филогенетический анализ показал, что таджикские изоляты Ehrlichia spp. очень близки с изолятами Ehrlichia spp., циркулирующими в Китае и Бразилии. Изолят Tajikistan-5 кластеризуется с предполагаемым новым видом Ehrlichia mineirensis. Таджик- ские изоляты Theileria spp. были генетически схожи с вариантами T. annulata, циркулирующими в Турции, Иране, Пакистане и Китае.

## Introduction

Ixodid ticks transmit various pathogens to both humans and animals in Asia (Tishkova et al., 2012; Wu et al., 2013). Twenty-three species of ixodid ticks have been described in this region of Central Asia, with the predominant ixodid tick species being Hyalomma anatolicum Koch, 1844 (Rasulov, 2007). The Crimean-Congo hemorrhagic fever, Sindbis, and Wad Medani viruses were previously detected in ixodid ticks in Tajikistan and other Asian countries (Begum et al., 1970; Gresíkova et al., 1978; Petrova et al., 2013). Hyalomma anatolicum ticks are also known to transmit bacterial and parasite infections such as Lyme disease, babesiosis, piroplasmosis, theileriosis, and anaplasmosis (Tishkova et al., 2012; Wu et al., 2013). Theileria annulata (Piroplasmida: Family Theileriidae, Genus Theileria) is the causative agent of theileriosis in domestic animals, which is transmitted by 15 species of ixodid ticks of the genus Hyalomma (Robinson, 1982). Ehrlichia spp. (Family Anaplasmataceae, Genus Ehrlichia) are intracellular Gram-negative bacteria, ecologically associated with ixodid ticks and their animal hosts (Parola et al., 2001). The pathogenicity to domestic and wild animals, as well as to humans, has been demonstrated in Ehrlichia canis, E. chaffeensis, E. ewingii, E. muris and E. ruminantium (Aguiar et al., 2014; Cabezas-Cruz et al., 2014). Currently, there are no published studies on genetic markers and genotyping of Rickettsia spp., Ehrlichia spp., Anaplasma spp., Babesia spp., and Theileria spp. in H. anatolicum ticks in Tajikistan.

The objectives of this study were to survey the prevalence of genetic markers for these tick-borne infections in H. anatolicum ticks collected in southwestern Tajikistan, and to perform sequence and phylogenetic analysis of Ehrlichia spp. and Theileria spp. detected in the ticks.

## Materials and methods

**Fig. 1.**
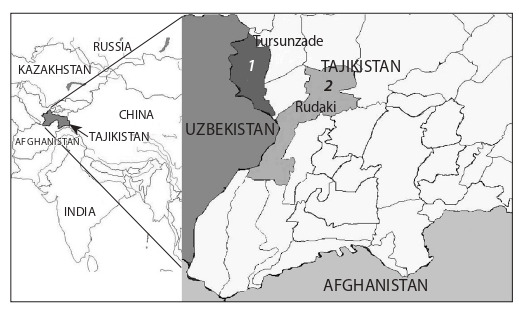
Tick collection sites in Tajikistan: 1, Tursunzade district; 2, Rudaki district.

**Tick harvesting.** Adult ticks were collected from domestic animals in several villages of the Rudaki district (Somoniyon N 38°26′27″, E 68°46′28″) and the Tursunzade district (Tursunzade N 38°30′39″, E 68°13′49″) in southwestern Tajikistan in July 2009 (Fig. 1). The ticks were transported and samples for analysis were prepared as described in (Petrova et al., 2013). Tick species were identified by morphological examination with subsequent confirmation by PCR and sequencing of PCR products of a 16S rRNA fragment of the mitochondrial genome of the ticks.

**PCR detection of genetic markers.** DNA was isolated from tick homogenates by phenol/chloroform extraction using a commercial kit (Lytech, Moscow, Russia) following manufacturer’s instructions. It was kept at −20 °C until use. The genetic markers of Rickettsia spp., Ehrlichia spp., Anaplasma spp., Babesia spp. and Theileria spp. in ticks were detected by PCR with specific primers (see the Table). The PCR fragments were purified using Wizard SV Gel and a PCR Clean-Up System kit (Promega, USA) according to manufacturer’s instructions. All PCR fragments were sequenced in a 3130 Genetic Analyzer automated capillary sequencer (Applied Biosystems Inc.). DNA sequencing reactions were performed with BigDyeTerminator v3.1 Cycle Sequencing Kits (Applied BioSystems, USA). Both strands of each gene fragment were directly sequenced; each sample was sequenced twice. Precautions were taken at all steps of analysis to avoid cross-contamination among samples.

**Table 1.**
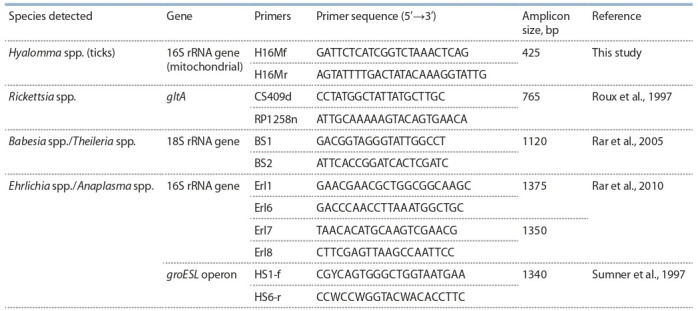
Primers used for PCR identification of ticks and tick-borne infections in the present study

**Nucleotide sequences and phylogenetic analyses.** DNA sequences were compared with sequences available in Gen- Bank using the Basic Local Alignment Search Tool (BLAST) on http://blast.ncbi.nlm.nih.gov. Evolutionary analyses were conducted with MEGA5 software (Tamura et al., 2011). Multisequence alignments were performed using ClustalX. For each analyzed gene a phylogram was constructed by the maximum likelihood method. Phylogenetic distances between homologous sequences were calculated using Kimura’s twoparameter model. Confidence levels for individual branches of the resulting tree were determined by bootstrap analysis with 1000 replicates.

## Results and discussion

**Tick harvesting**

Adult H. anatolicum ticks (138 females and 244 males) were collected and grouped in 137 pools. Tick species were identified by sequencing a fragment of 16S rRNA mitochondrial gene for all pools. Two original variants of 16S rRNA mitochondrial gene fragment sequences found in these ticks were submitted to GenBank (accession numbers KP059123 and KP059124). The nucleotide fragments showed 99.9 % similarity to the corresponding H. anatolicum sequences from GenBank. These tick pools were tested by PCR for genetic markers of Rickettsia spp., Ehrlichia spp., Anaplasma spp., Babesia spp., and Theileria spp. and other ticks were used for genotyping. Of those ticks, 290 (179 males and 111 females) were collected in the Tursunzade district and 92 (65 males and 27 females) from the Rudaki district. The PCR tests for Ehrlichia spp. and Theileria spp. were positive in the range 0.7–3.3 %. The PCR tests for Rickettsia spp., Anaplasma spp., and Babesia spp. were negative in all tick samples.

**Theileria identification**

**Fig. 2.**
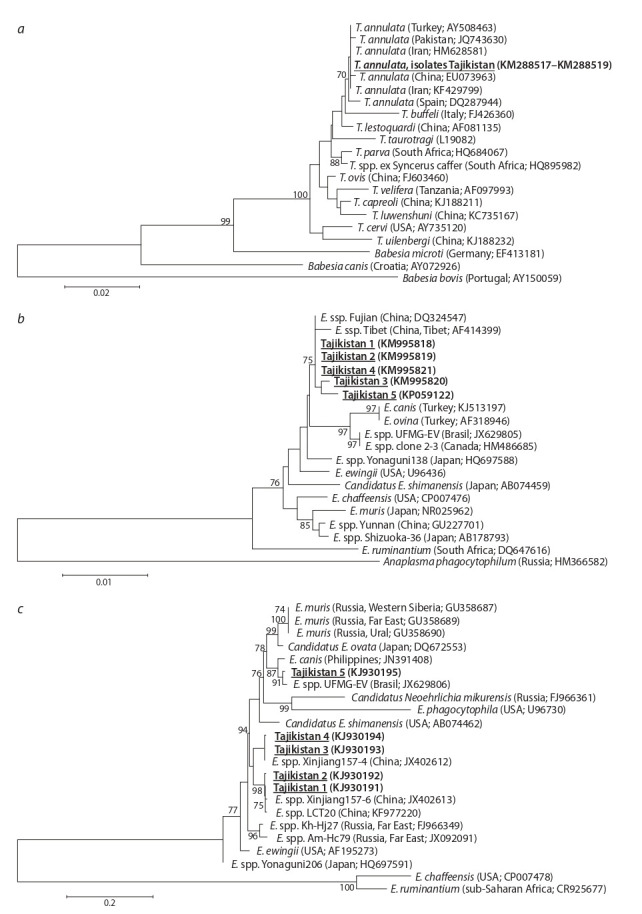
Phylogenetic tree of Theileria spp. and Ehrlichia spp. isolates: a, based on 18S RNA of Theileria spp.; b, based on the 16S rRNA gene of Ehrlichia spp.; c, based on the groESL operon of Ehrlichia spp. For each gene analyzed, a phylogram was constructed by the maximum likelihood method. Phylogenetic distances between homologous sequences were calculated using Kimura’s two-parameter model. Confidence values for individual branches of the resulting tree were determined by bootstrap analysis with 1000 replicates.

Theileria spp. was detected in 3.3 % ticks from Rudaki but not in ticks from Tursunzade. The amplified PCR fragments of 18S rRNA (1090–1092 bp) were isolated and sequenced (GenBank accessions KM288517–KM288519). The sequences were 100 % identical to isolates of Theileria annulata circulating in Turkey (AY508463) and Iran (KF429799, HM628581), similar by 99.9 % to isolated from Pakistan (JQ743630) and China (EU073963) and by 99.7 % to isolates from Spain (DQ287944). Phylogenetic analysis confirmed that Theileria spp. isolates from the Rudaki district of Tajikistan belonged to Th. annulata (Fig. 2). The analysis of 18S rRNA gene fragment for three isolates of Th. annulata from southwestern Tajikistan showed that all isolates were genetically identical (100 % similarity).

**Ehrlichia identification**

The presence of Ehrlichia spp. has not been previously documented in ticks and animals in Tajikistan. DNA of Ehrlichia spp. was detected in five pools of H. anatolicum ticks collected in the Rudaki and Tursunzade districts. The infection rates for Ehrlichia spp. were 3.3 % in Rudaki and 0.7 % in Tursunzade. The fragments of the 16S rRNA gene (1291– 1352 bp) and groESL operon (1248–1315 bp) were sequenced (KM995818–KM995821, KP059122, KJ930191–KJ930195). The nucleotide sequences of 16S rRNA gene fragments were highly conserved (99.5–100 %) among studied isolates. The similarity levels of the studied 16S rRNA fragments to E. chaffeensis (CP007478), E. canis (KJ513197), and E. muris (NR121714) were 99.2, 99.2, and 99.3 %, respectively.

The phylogenetic tree generated using Ehrlichia spp. groESL operon fragment sequences was markedly different from the tree based on 16S rRNA sequences (Fig. 2, b, с). The 16S rRNA gene fragment analysis (1140 nucleotides) showed that all isolated Ehrlichia spp. were genetically close (see Fig. 2, b). The studied isolates grouped in the same branch of the phylogenetic tree as isolates from the Fujian province in Southeastern China (DQ324547) and the Tibet Autonomous Region of China (AF414399). The Tibetan isolate was grouped with the E. canis branch, which is genetically close to the species E. chaffeensis, pathogenic for humans (Wen et al., 2002). We note that the Tajik isolates were most similar to Chinese isolates from regions of China that do not border Tajikistan. The phylogenetic tree generated using Ehrlichia spp. groESL operon fragment sequences was markedly different from the tree based on 16S rRNA sequences (see Fig. 2, c). The nucleotide sequences of groESL operon Ehrlichia spp. found in Tajikistan are separated into three groups. Tajikistan 1 and 2 isolates were closest to two isolates Ehrlichia spp. from different regions of China (Xinjiang, Hyalomma asiaticum; Yunnan, Rhipicephalus microplus), Tajikistan 3 and 4 cluster with a different Chinese isolate (Xinjiang, Hyalomma asiaticum). Tajikistan 5 showed high similarity to Ehrlichia spp. (JX629806) isolated in Brazil from a Rhipicephalus microplus tick (Cruz et al., 2012). Tajikistan 5 has 13 nucleotide and 2 amino acid substitutions in comparison to the Brazilian isolate. The American isolate was previously identified as a new species of Ehrlichia spp. named E. mineirensis. It causes clinical manifestations associated with ehrlichiosis in experimentally infected calf (Aguiar et al., 2014).

Tajikistan 1–4 isolates clustered with Chinese isolates from Xinjiang and Yunnan Provinces. Xinjiang Province shares borders with Tajikistan in southwestern China, unlike Yunnan. Tajikistan 5 isolate was the most genetically distinct from other Ehrlichia spp. grouping with UFMG-EV and UFMTBV isolates from Brazil and BOV2010 isolate from Canada (Gajadhar et al., 2010; Aguiar et al., 2014; Cabezas-Cruz et al., 2014). We infer that Tajikistan 5 isolate belongs to the putative novel species of Ehrlichia spp. previously named E. mineirensis.

## Conclusions

Hyalomma anatolicum ticks collected in Tajikistan were tested by PCR for markers of tick-borne bacterial and protozoan infections. DNA of Ehrlichia spp. and Theileria spp. was detected in ticks collected from the Rudaki and Tursunzade districts. The infection rates for Ehrlichia spp. and Theileria spp. DNA markers ranged within 0.7–3.3 % according to PCR. Fragments of the 16S rRNA gene and groESL operon from Ehrlichia spp. and of the 18S rRNA gene from Theileria spp. were isolated and sequenced from H. anatolicum ticks. Phylogenetic analysis demonstrated that Ehrlichia spp. isolates were highly similar to Ehrlichia spp. circulating in China and Brazil. Isolate Tajikistan 5 was closely related to the putative novel species E. mineirensis. The Tajik isolates of Theileria spp. were genotyped as Theileria annulate, and fragments of the 18S rRNA gene from these isolates were highly similar to the 18S rRNA gene of T. annulata isolates from Turkey, Iran, Pakistan and China.

## Conflict of interest

The authors of this study have no commercial associations that might create a conflict of interest to the present work. All authors are working in non-profit federal organizations. No competing financial interests exist.
